# Editorial: Proteomic and metabolic reprogramming in myeloma cells within the tumor microenvironment

**DOI:** 10.3389/fonc.2023.1264740

**Published:** 2023-08-07

**Authors:** Enrico Milan, Annamaria Gullà

**Affiliations:** ^1^ Age Related Diseases Unit, Division of Genetics and Cell Biology, San Raffaele Scientific Institute, Milano, Italy; ^2^ Faculty of Medicine and Surgery, University Vita-Salute San Raffaele, Milano, Italy; ^3^ Experimental Hematology and Immunology, Candiolo Cancer Institute, FPO-IRCCS, Candiolo, Italy; ^4^ Department of Medical Oncology, Dana Farber Cancer Institute, Boston, MA, United States

**Keywords:** multiple myeloma, bone marrow, metabolism, mitochondria, proteasome inhibitors (PI), IMiDs, microenvironment

Multiple myeloma (MM) is a hematological malignancy characterized by the dysregulated proliferation of transformed plasma cells (PCs) primarily in the bone marrow (BM) (1). For the highly specialized role as antibody secreting factories, PCs and their malignant counterpart must cope with different stressors linked to massive immunoglobulin synthesis (2). As such, they display an exquisite dependency on stress adaptive, protein degradative and metabolic pathways that ensure the sustainability of such a demanding process (3–5). While providing survival advantage, these “lineage” dependencies have similarly highlighted fundamental therapeutic vulnerabilities that have led to the introduction of the proteasome inhibitor (PI) bortezomib in the clinical setting (6–9). On the other hand, the strict interaction of MM cells with the immune and non-immune components of the BM milieu is a pathognomonic feature of the disease (10); and it is becoming clear how metabolic and proteomic reprogramming in MM cells can shape this mutual interaction, thus favoring a protective and MM-promoting environment (11, 12). The clinical success of immunomodulatory agents (IMiDs) and immunotherapy that alter the tumor-BM interface underscores the importance of its targeting (13–17), prompting for a deeper understanding of the role of metabolic rewiring in this interaction to unveil novel therapeutic dependencies. In this scenario, the reviews and original articles of this Research Topic give insights on open questions on the tumor-intrinsic or tumor-host mechanisms driving disease progression, emphasizing the emerging role of metabolic dependencies as new potential therapeutic opportunities.

Specifically, two reviews discuss the role of the MM-*milieu* interplay in mediating resistance to PIs (Schwestermann et al.) and IMiDs (Chen and Gooding). While providing a detailed summary of the current knowledge on the interaction between MM cells and BM components, Schwestermann et al. have focused on how the metabolic rewire shapes MM cell ability to adapt, grow and resist to therapies, specifically PIs. Importantly, they highlight how metabolic switches occurring after treatment with PIs may be targeted to overcome PI-resistance and improve overall drug efficacy. On a similar line, Chen and Gooding summarize the current literature on the mechanisms underlying IMiDs resistance with a specific highlight at the gap existing in the characterization of Cereblon (CRBN)-independent mechanisms, including those related to immune microenvironment and metabolism. In a broader light, Solimando et al. provide an extensive overview on the mechanisms by which MM resist to different therapies, including epigenetic modification and cancer cell stemness; and discuss therapeutic strategies to improve the management of MM patients.

Of clinical relevance, Raimondi et al. discuss the importance of metabolic reprogramming of MM cells in the context of MM-related bone disease. Specifically, they focus on the role of glutamine addiction, in controlling osteoblast differentiation from mesenchymal stromal cells, and thus bone remodeling. While providing an exhaustive review of the implication of metabolic features in the pathophysiology of MM bone disease, they also emphasize the clinical implication of such findings that may allow for the identification of new tracers of active bone disease that are under investigation that may be more sensitive in patients with negativity at the PET/TC with ^18^F-fluorodeoxyglucose (FDG).

The local concentration of metabolites in the BM milieu may affect MM progression and drug sensitivity (12). The original article by Trudu et al. in this Research Topic, describes a novel intrinsic role of arginine shortage in directly promoting MM proliferation and resistance to therapies. The authors show that, beyond the established immunosuppressive role of arginine deprivation on T cells (18), the reduction in arginine availability may have a direct pro-survival effect, mediated by increased activation of AKT, that results in higher PI-resistance to *in vitro* and MM growth *in vivo*.

Growing evidence are showing that mitochondria are critical for MM development, progression, and sensitivity to therapies. Two reviews in this Research Topic analyzed the role of mitochondrial metabolism in general in MM pathobiology (Nair et al.), or in particular by focusing on the MM tumor suppressor TRAF3 (Jung et al.) a signal transducer that has been recently showed to be recruited to the mitochondria in B cells (19). Nair et al. extensively analyze how mitochondria metabolism influences MM development and drug resistance and *vice versa* how MM mutations affect metabolic pathways. The authors also discuss the role of mitochondria in regulating MM metabolism, redox homeostasis, iron and calcium fluxes, and the activation of the integrated stress response and intrinsic apoptosis. On this line, Jung et al., describe the role of TRAF3 in normal and malignant B cells. TRAF3 is a negative regulator of key B cell signaling pathways, whose loss induces enhanced antibody responses and increased B cell survival (20, 21). Moreover, recent findings have shown that it is recruited to the mitochondrial membrane through its interaction with the mitochondrial fission factor MFF (19). In their mini-review, the authors summarize the current knowledge on TRAF3 signaling activities and on these newly discovered roles in modulating mitochondrial morphology, cellular metabolism, and mitochondria-dependent apoptosis.

Finally, novel original culture methods for the analysis of the interplay between BM adipocyte-MM cells have been presented by Fairfield et al. The authors propose three *in vitro* culture systems allowing the study of MM-adipocyte crosstalk using primary BM adipocyte and not those obtained through the differentiation *in vitro* of BM-derived mesenchymal stromal cells. Of technological relevance, the authors describe in detail the protocols to perform 2D and 3D co-cultures and the following imaging and proteomic characterizations, providing novel tools to dissect how adipocytes and MM cells influence each other in the BM environment.

In summary, this Research Topic provides a critical overview and novel evidence on the role of proteomic and metabolic reprogramming of myeloma cells as key drivers of MM pathogenesis and drug resistance with important therapeutic implications. Open questions remain on how protein degradative and metabolic pathways affect immune activation and tumor sensitivity to immunotherapies. Further investigations will be instrumental in exploiting such metabolic and proteomic dependencies to improve the therapeutic efficacy of anti-MM agents, increase response durability and patient overall outcome.

## Author contributions

EM: Writing – original draft, Writing – review & editing, AG: Writing – original draft, Writing – review & editing.
